# Strong Agreement of Nationally Recommended Retention Measures from the Institute of Medicine and Department of Health and Human Services

**DOI:** 10.1371/journal.pone.0111772

**Published:** 2014-11-06

**Authors:** Peter F. Rebeiro, Michael A. Horberg, Stephen J. Gange, Kelly A. Gebo, Baligh R. Yehia, John T. Brooks, Kate Buchacz, Michael J. Silverberg, John Gill, Richard D. Moore, Keri N. Althoff, for the North American AIDS Cohort Collaboration on Research and Design (NA-ACCORD)

**Affiliations:** 1 Johns Hopkins University, Baltimore, MD, United States of America; 2 Kaiser Permanente Mid-Atlantic States, Rockville, MD, United States of America; 3 University of Pennsylvania Perelman School of Medicine, Philadelphia, PA, United States of America; 4 Centers for Disease Control and Prevention, Atlanta, GA, United States of America; 5 Kaiser Permanente Northern California, Oakland, CA, United States of America; 6 University of Calgary, Calgary, AB, Canada; Infectious Disease Service, United States of America

## Abstract

**Objective:**

We sought to quantify agreement between Institute of Medicine (IOM) and Department of Health and Human Services (DHHS) retention indicators, which have not been compared in the same population, and assess clinical retention within the largest HIV cohort collaboration in the U.S.

**Design:**

Observational study from 2008–2010, using clinical cohort data in the North American AIDS Cohort Collaboration on Research and Design (NA-ACCORD).

**Methods:**

Retention definitions used HIV primary care visits. The IOM retention indicator was: ≥2 visits, ≥90 days apart, each calendar year. This was extended to a 2-year period; retention required meeting the definition in both years. The DHHS retention indicator was: ≥1 visit each semester over 2 years, each ≥60 days apart. Kappa statistics detected agreement between indicators and C statistics (areas under Receiver-Operating Characteristic curves) from logistic regression analyses summarized discrimination of the IOM indicator by the DHHS indicator.

**Results:**

Among 36,769 patients in 2008–2009 and 34,017 in 2009–2010, there were higher percentages of participants retained in care under the IOM indicator than the DHHS indicator (80% vs. 75% in 2008–2009; 78% vs. 72% in 2009–2010, respectively) (p<0.01), persisting across all demographic and clinical characteristics (p<0.01). There was high agreement between indicators overall (κ = 0.83 in 2008–2009; κ = 0.79 in 2009–2010, p<0.001), and C statistics revealed a very strong ability to predict retention according to the IOM indicator based on DHHS indicator status, even within characteristic strata.

**Conclusions:**

Although the IOM indicator consistently reported higher retention in care compared with the DHHS indicator, there was strong agreement between IOM and DHHS retention indicators in a cohort demographically similar to persons living with HIV/AIDS in the U.S. Persons with poorer retention represent subgroups of interest for retention improvement programs nationally, particularly in light of the White House Executive Order on the HIV Care Continuum.

## Introduction

Retention in clinical care for HIV-infected patients is important for achieving and maintaining improved individual and public health outcomes [1], [2]. The Institute of Medicine (IOM) and the US Department of Health and Human Services (DHHS) recently endorsed two different indicators for retention in HIV care. The IOM indicator, similar to one proposed by the Health Resources and Services Administration (HRSA) HIV/AIDS Bureau in 2009, summarizes clinical retention across a 12-month period [3]. The DHHS indicator requires a 24-month period to measure retention, which is consistent with the current HRSA guidelines (altered in 2013) [4], [5]. Because of the potential for adoption of “competing” standards by different agencies or research groups, we undertook a comparison of the IOM and DHHS retention-in-care metrics [6], [7] using data from the North American AIDS Cohort Collaboration On Research and Design (NA-ACCORD).

## Methods

### Study population

**Figure 1:**
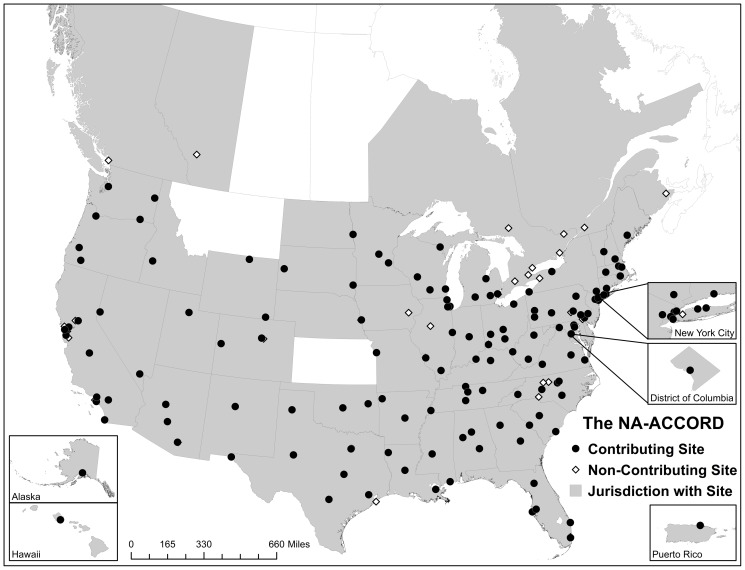
Geographic distribution of North American AIDS Cohort Collaboration on Research and Design (NA-ACCORD) clinical sites contributing to these analyses. Non-contributing sites were interval cohorts, Canadian cohorts (excluded due to the focus on US clinical care populations), or cohorts not currently contributing HIV primary care encounter data to the NA-ACCORD.

The NA-ACCORD is the largest multi-site collaboration of interval and clinic-based cohort studies of HIV-infected adults (≥18 years old) receiving care in the U.S. and Canada [8], [9]. We conducted serial, annual cross-sectional analyses using data contributed to NA-ACCORD U.S. clinical cohorts by participants who had ≥1 HIV primary care visit between January and July of 2008 or of 2009. This allowed us to focus on retention in clinical care according to both the IOM and DHHS definitions in the period of January 2008 to December 2010 [3], [4], [5]. The eleven included cohorts had clinical sites in all 50 U.S. states, Washington, D.C., and Puerto Rico (Figure 1). Participant written consent or else a waiver of consent was obtained and documented by each cohort site with the approval of the local IRB. All data were de-identified locally before being transmitted and harmonized at a central Data Management Core. The activities of the NA-ACCORD have been reviewed and approved by the local institutional review boards (IRB) for each site and at Johns Hopkins School of Medicine.

### Outcomes

Retention in clinical care was assessed in 2008–2009 and 2009–2010 using two different indicators defined as: 1) IOM-endorsed: the numerator was the number of adults with ≥2 HIV primary care visits within each calendar year, >90 days apart, and the denominator was adults with ≥1 visit during the year; 2) DHHS-endorsed: the numerator was the number of adults with an HIV primary care visit in each semester (January–June and July–December) of the 2-year period, each visit ≥60 days after the prior, and the denominator was adults with ≥1 visit during the first semester of the first year. The IOM-based definition was extended to a 2-year period (2008–2009 and 2009–2010) to allow direct comparison with the DHHS-based definition; patients were considered retained only if they met the numerator inclusion requirements in both years, and the denominator was restricted to adults with ≥1 visit in the first semester of the first year. Inpatient visits were excluded.

### Demographic and clinical characteristics analyzed for differences

We investigated whether clinical retention was associated with demographic characteristics, including age at first visit during the study period, race/ethnicity, HIV transmission risk group, sex, CD4+ lymphocyte (CD4) count, prescription of antiretroviral therapy (ART) during the study period, and suppression of plasma HIV-1 RNA. Race/ethnicity was categorized as non-Hispanic black, non-Hispanic white, Hispanic, and other/unknown. HIV transmission risk group was categorized as men who have sex with men (MSM), injection drug use (IDU), heterosexual contact, and other/unknown. Patients with both sexual and IDU transmission risk were categorized as IDU. CD4 count at baseline was categorized as <350 cells/µL, 350–499 cells/µL, or ≥500 cells/µL. ART was defined as a regimen of >3 antiretroviral agents from ≥2 classes or a triple nucleoside/nucleotide reverse transcriptase inhibitor (NRTI) regimen containing abacavir or tenofovir (consistent with contemporary guidelines) [10]. Patients were classified as using ART if ART was prescribed for ≥6 months in a calendar year. HIV-1 RNA suppression was defined as plasma HIV-1 RNA <200 copies/mL, and was assessed at last measure in the study period, per the DHHS indicator for HIV-1 RNA suppression [4], [5].

### Statistical Analysis

Chi-square tests were used to identify differences between the two indicators by demographic and clinical characteristics; kappa statistics were used to detect agreement between indicators. C statistics (areas under Receiver-Operating Characteristic curves) from logistic regression analysis were used to summarize “correct” classification of patients as retained or not by the DHHS indicator (using the IOM indicator as the reference standard); a sensitivity analysis using the DHHS indicator as the reference standard was also conducted [11]. The logistic regression accounted for clustering of outcomes among individuals across the 2008–2009 and 2009–2010 periods and utilized robust variances.

## Results

During 2008–2009 and 2009–2010, there were significantly higher percentages of participants retained in care under the IOM indicator (80% = 29,377/36,769 in 2008–2009; 78% = 26,522/34,017 in 2009–2010) than the DHHS indicator (75% = 27,635/36,769 in 2008–2009; 72% = 24,334/34,017 in 2009–2010) (p<0.01). This difference persisted, with higher percentages retained by IOM than by DHHS indicator, across all demographic and clinical characteristics measured in both time periods (p<0.01, Table 1).

**Table 1:** Adults retained in clinical care in the NA-ACCORD, according to demographic and clinical characteristics, 2008–2010.

Characteristic	2008–2009					2009–2010				
	(% of N = 36,769)	% Retained in Care by IOM indicator	% Retained in Care by DHHS indicator	Kappa Statistic^a^ (Agreement)	C Statistic^b^ (Predicted IOM by DHHS)	(% of N = 34,017)	% Retained in Care by IOM indicator	% Retained in Care by DHHS indicator	Kappa Statistic^a^ (Agreement)	C Statistic^b^ (Predicted IOM by DHHS)
**Total**	**(100)**	**80**	**75**	**0.83**	**0.960**	**(100)**	**78**	**72**	**0.79**	**0.947**
**Age (years)**					**0.956**					**0.939**
≤39	**(17)**	**70**	**64**	**0.82**		**(14)**	**71**	**65**	**0.83**	
40–49	**(33)**	**78**	**73**	**0.82**		**(31)**	**76**	**69**	**0.80**	
50–59	**(33)**	**83**	**78**	**0.83**		**(35)**	**80**	**73**	**0.77**	
≥60	**(18)**	**87**	**84**	**0.84**		**(21)**	**83**	**77**	**0.76**	
**Sex**					**0.951**					**0.936**
Male	**(84)**	**80**	**75**	**0.83**		**(84)**	**78**	**71**	**0.79**	
Female	**(16)**	**80**	**75**	**0.83**		**(16)**	**80**	**74**	**0.80**	
**Race/Ethnicity**					**0.952**					**0.935**
Non-Hispanic White	**(42)**	**80**	**76**	**0.83**		**(41)**	**78**	**71**	**0.79**	
Non-Hispanic Black	**(43)**	**80**	**75**	**0.83**		**(44)**	**78**	**71**	**0.78**	
Hispanic	**(11)**	**81**	**76**	**0.82**		**(11)**	**83**	**78**	**0.82**	
**HIV Risk Group**					**0.952**					**0.937**
MSM	**(32)**	**78**	**73**	**0.82**		**(32)**	**78**	**73**	**0.84**	
IDU	**(18)**	**80**	**75**	**0.84**		**(18)**	**78**	**70**	**0.75**	
Hetero	**(21)**	**79**	**73**	**0.83**		**(21)**	**80**	**74**	**0.81**	
**CD4+ Count^c^**					**0.952**					**0.939**
≤349 (cells/µL)	**(27)**	**76**	**70**	**0.82**		**(26)**	**76**	**69**	**0.79**	
350–499 (cells/µL)	**(19)**	**82**	**77**	**0.82**		**(20)**	**81**	**75**	**0.78**	
≥500 (cells/µL)	**(38)**	**83**	**79**	**0.83**		**(42)**	**81**	**75**	**0.78**	
**ART^d^**					**0.957**					**0.944**
No Prescription	**(29)**	**62**	**54**	**0.82**		**(26)**	**59**	**50**	**0.78**	
Prescription	**(71)**	**87**	**84**	**0.81**		**(74)**	**85**	**79**	**0.76**	
**HIV RNA^e^**					**0.951**					**0.936**
≥200 (copies/mL)	**(26)**	**73**	**67**	**0.83**		**(24)**	**72**	**65**	**0.80**	
<200 (copies/mL)	**(64)**	**85**	**81**	**0.81**		**(66)**	**82**	**76**	**0.77**	

Retention defined by the Institute of Medicine's and Department of Health and Human Services' retention indicators.

DHHS: Department of Health and Human Services; Hetero: heterosexual contact; IDU: injection drug use; IOM: Institute of Medicine; MSM: male sexual contact with men.

DHHS Indicator: ≥1 visit in each semester (January–June or July–December), >60 days apart, over a 2-year period.

IOM Indicator: ≥2 visits in each calendar year, >90 days apart, over a 2-year period (this definition was extended from 1 to 2 years for direct comparison with the DHHS indicator).

a: all different from 0, p<0.01;

b: Area under receiver-operating characteristic curves resulting from logistic models accounting for clustering across two years with robust variances adjusted for respective covariates, with “Total” category adjusted for all covariates.

c: At first measurement in 2008 or 2009; d: For ≥6 months in 2008 and 2009 or in 2009 and 2010; e: At last measurement in 2009 or 2010.

All characteristics differed different by the percentages retained across different indicators, χ^2^ p<<0.001. Percentages may not sum to 100 due to rounding or missing values.

Across demographic characteristics, the percentage of patients who met clinical retention by the IOM and DHHS definitions varied from 62–87% and 54–84%, respectively in 2008–2009, and from 59–85% and 50–79%, respectively in 2009–2010. Regardless of definition or year, the lowest percentages retained in care were seen among persons aged ≤39 years, persons not prescribed ART≥6 months in each year, and persons with unsuppressed HIV-1 RNA (Table 1). Persons not prescribed ART≥6 months in each year were retained in significantly lower proportions than by any other characteristic in both time periods, though these individuals were a minority of the sample (29% in 2008–2009 and 26% in 2009–2010).

There was very high agreement between indicators overall (94% agreement and κ = 0.83 in 2008–2009; 92% agreement and κ = 0.79 in 2009–2010, p<0.001). Strong agreement in the IOM and DHHS indicators persisted across all demographic and clinical characteristics examined (κ≥0.81, p<0.01 for κ≠0 in all comparisons in 2008–2009; κ≥0.75, p<0.01 in 2009–2010; Table 1). C statistics revealed a very strong ability to predict retention according to the IOM indicator based on the DHHS indicator status in both unadjusted and adjusted analysis (Table 1); similarly, the IOM indicator was also a strong predictor of retention under the DHHS definitions (data not shown).

## Discussion and Conclusions

Clinical retention by the IOM indicator was 5–6% higher compared with retention by the DHHS indicator during the two study periods, although strong agreement exists between these metrics. For policy and monitoring purposes, the DHHS indicator is more conservative due to restrictions on when visits must occur and excludes individuals from measurement if they entered care in the latter three-quarters of the 24-month measurement window; however, the IOM indicator is better suited for assessing retention over longer periods of time.

The lower percentage retained in care by the DHHS indicator is a direct result of the DHHS indicator's stringent requirements for the timing of visits in different semesters across the 2-year period: a visit in each and every semester of the 2-year period of observation (>60 days apart) was required to be classified as retained in care.

The denominator requirements for the DHHS indicator may induce intra-individual missingness of retention outcomes across >24-month intervals. This is because a patient may be included in the denominator during the first 24-month window and subsequently excluded as the window “moves” forward, if they have no qualifying visit in the initial semester of the new 24-month interval. This was the case for 8.3% of individuals who were eligible to be included in the DHHS indicator denominator only in 2008–2009 (and not in 2009–2010), despite qualifying for the unaltered IOM indicator in both 12-month time periods.

By contrast, the IOM numerator included individuals with any 2 visits (>90 days apart) during each calendar year (whether in different semesters or not). Although the IOM indicator is defined for a 12-month period, we employed a 24-month period by requiring the IOM indicator definition to be met in both calendar years of the 24-month period to allow for a more direct comparison with the 24-month period articulated in the DHHS indicator definition.

The IOM indicator may be more desirable than the DHHS indicator when assessing clinical retention within individuals over longer periods of time. Both indicators revealed the same groups in need of targeted intervention to improve retention; namely the young, and those not prescribed ART. These characteristics allude to the challenge of engaging HIV-infected adults in care when they may not yet be feeling the effects of their HIV infection.

The application of these indicators in different populations needs to be refined as clinical practice and guidelines for laboratory monitoring frequency change. Stable patients with suppressed HIV-1 RNA viral loads and high CD4+ cell counts may require fewer annual primary care visits and laboratory tests than patients with poorer health status; the utility of retention indicators to highlight gaps in the continuum of care related to negative individual- and population-level HIV outcomes should consequently be re-examined as guidelines are revised over time.

Limitations to this analysis include possible difficulties in generalizing the results to populations of HIV-infected individuals newly linked to care. The study population was successfully engaged in care; about 93% were in care prior to 2008. It has been noted that nearly 33% of individuals newly linked to care are not retained in care in the United States [1], [12]. If there are differences in retention measured by these indicators in the period following initial linkage to care, or in any period where the proportion retained is very low, they may not be detected here. In addition, the association of these results with clinical outcomes and potential changes in the results produced by changing clinical guidance regarding the frequency of clinical monitoring should be studied longitudinally to further characterize the utility of each metric.

Application of both the IOM and DHHS indicators yielded fairly similar results and showed strong agreement. These results should provide reassurance that utilizing the IOM indicator for annual or cross-sectional assessments at the clinic level should not produce widely disparate results compared with the DHHS approach [13]. Further, while clinical retention was high across most demographic and clinical subsets, our analysis highlights that specific subgroups could benefit from enhanced clinical retention efforts: younger individuals and persons not prescribed ART. These groups should be the focus of clinicians and policy makers as they seek to implement the National HIV/AIDS Strategy, with the understanding that even in a population of individuals successfully linked to care after diagnosis, significant challenges may remain in maintaining clinical retention.
